# Characteristics of measurable residual disease assessment in myeloma: a review of clinical trials from 2015–2020

**DOI:** 10.1038/s41408-022-00750-1

**Published:** 2022-11-15

**Authors:** Oliver Van Oekelen, Nicole Birrer, William Wesson, Vincent L. Galate, Edward R. Scheffer Cliff, Aaron M. Goodman, Al-Ola Abdallah, Rajshekhar Chakraborty, Vinay Prasad, Ghulam Rehman Mohyuddin

**Affiliations:** 1 Department of Internal Medicine, Icahn School of Medicine at Mount Sinai, Mount Sinai Beth Israel, Department of Internal Medicine, New York, NY USA; 2 University of Utah, Huntsman Cancer Center, Division of Hematology, Salt Lake City, Utah USA; 3 University of Kansas, University of Kansas School of Medicine, Kansas City, Kansas USA; 4 Program on Regulation, Therapeutics and Law, Brigham and Women’s Hospital, Harvard Medical School, Boston, MA USA; 5 Division of Blood and Marrow Transplantation, University of California San Diego, San Diego, CA USA; 6 Department of Hematology, Columbia University, New York, NY USA; 7 Division of Hematology/Oncology, Department of Medicine, University of California San Francisco, San Francisco, CA USA

**Keywords:** Myeloma, Clinical trials

**To the Editor:**

As the survival of patients with newly diagnosed multiple myeloma (MM) has continued to improve, there is a need for prolonged follow-up to demonstrate a progression-free or overall survival benefit [1]. A valid surrogate endpoint could reduce cost and duration of trials, and thus accelerate access to effective treatments [2]. Novel treatments have resulted in deep serological responses and modern sensitive methods use either next-generation flow cytometry (NGF) or next-generation sequencing (NGS) to detect malignant plasma cells in the bone marrow. This measurable (or minimal) residual disease (MRD) assessment has been suggested to improve the sensitivity of response evaluation and has been proposed as a surrogate for progression-free survival (PFS) in myeloma [3, 4].

Multiple studies have shown that stratification of patients by MRD status is associated with improved PFS [4–7]. In these contexts, MRD negativity is a clear prognostic marker. However, widespread implementation is limited by heterogeneity of the method, sensitivity, and timing of MRD evaluation.

The landscape of MRD assessment in myeloma clinical trials has not been comprehensively reported. In this report we aim to describe the implementation of MRD in clinical trials of MM between 2015 and 2020 by characterizing trials that utilize MRD. A previously used dataset and search strategy, as reported by Wesson et al. was utilized [8]. The query was performed on February 20, 2021, and data was collected between February 20, 2021, and May 31, 2022. We included all interventional trials of myeloma with a trial start date between January 1st, 2015, and December 31st, 2020. Trials that were terminated early after having enrolled patients, and that were not yet enrolling at time of data collection were included to best represent the trial landscape available to patients. Observational or non-interventional trials and studies involving non-plasma cell malignancies were excluded. We opted to use ClinicalTrials.gov as it allows querying ongoing studies for which data has not been published or reported otherwise. For clinical trials that included MRD as part of the study description in ClinicalTrials.gov, a manual search was conducted to determine whether data and protocols of MRD assessment were available publicly. Search terms included the National Clinical Trial number and the title of the study. Our search was conducted by two independent authors whose assessment was compiled into one central database. Conflicts regarding trial characteristics were discussed with an independent third author.

The primary aim was to determine the proportion of trials that collected MRD data within the ClinicalTrials.gov database. Secondary aims were to study the proportion of trials that used MRD as part of the inclusion criteria, as a primary, secondary and/or exploratory endpoint, and as a stratification tool to determine treatment, while also examining the sensitivity and method of MRD assessment, and to characterize how use of MRD assessment in clinical trial protocols has changed over time. Comparison of studies assessing MRD to those that do not was conducted using the Fisher’s exact test. A two-sided *p* value of less than 0.05 was considered significant. All statistical analyses were conducted in R (v4.0.2).

A total of 598 MM studies were included (Supplementary Table 1, Supplementary Fig. 1), the majority of which included chemotherapy (65.2%, *n* = 390), were phase 1 or 2 (30.4%, *n* = 182 and 36.1%, *n* = 216 respectively), and recruited relapsed/refractory MM (61.0%, *n* = 365) (Table 1). MRD assessment was reported as being a part of the trial in ClinicalTrials.gov for 145 of these studies (24.2%). Of these, 37.9% (*n* = 55) were randomized trials and 10.3% (*n* = 15) had MRD status at enrollment as part of the inclusion criteria. Most studies (92.4%, *n* = 134) included MRD assessment as an endpoint, most commonly (62.1%, *n* = 90) as a secondary endpoint. MRD status was a part of the primary endpoint (either by itself or as a co-primary endpoint) in 24.8% (*n* = 36) of studies. Notably, only 9 of the 145 trials (6.2%) utilized MRD assessment as a stratification tool to determine treatment.

**Table 1:** Characteristics of all included study trials.

	All studies		MRD measured		MRD not measured		*p* value
	*n* = 598		*n* = 145		*n* = 453		
Type of intervention							<0.001
Cell therapy	89	14.9%	16	11.0%	73	16.1%	0.14
Chemotherapy	390	65.2%	102	70.3%	288	63.6%	0.16
Combination	58	9.7%	25	17.2%	33	7.3%	0.001
Procedure	20	3.3%	2	1.4%	18	4.0%	0.18
Supportive Care	41	6.9%	0	0.0%	41	9.1%	<0.001
Phase of clinical trial							<0.001
Phase 1	182	30.4%	18	12.4%	164	36.2%	<0.001
Phase 1/2	89	14.9%	18	12.4%	71	15.7%	0.42
Phase 2	216	36.1%	73	50.3%	143	31.6%	<0.001
Phase 3	65	10.9%	33	22.8%	32	7.1%	<0.001
Phase 4	13	2.2%	1	0.7%	12	2.6%	0.21
Not Applicable	33	5.5%	2	1.4%	31	6.8%	0.011
Study population reported							0.18
Adult	572	95.7%	142	97.9%	430	94.9%	0.11
Geriatric	17	2.8%	3	2.1%	14	3.1%	0.77
Both	9	1.5%	0	0.0%	9	2.0%	/
Disease stage							<0.001
Newly diagnosed	116	19.4%	48	33.1%	68	15.0%	<0.001
Relapsed/Refractory	365	61.0%	67	46.2%	298	65.8%	<0.001
Both	75	12.5%	14	9.7%	61	13.5%	0.25
Maintenance	40	6.7%	16	11.0%	24	5.3%	0.022
Other	2	0.3%	0	0.0%	2	0.4%	1
Study sponsor							0.427
Industry	382	63.9%	97	66.9%	285	62.9%	
Non-industry	216	36.1%	48	33.1%	168	37.1%	
Location							0.015
United States (US)	265	44.3%	58	40.0%	207	45.7%	
Non-US	236	39.5%	52	35.9%	184	40.6%	
Multi-center including US	97	16.2%	35	24.1%	62	13.7%	
Primary study site location							0.002
Developed Country	489	81.8%	131	90.3%	358	79.0%	
Developing Country	109	18.2%	14	9.7%	95	21.0%	
Randomization							<0.001
Non-randomized	441	73.7%	90	62.1%	351	77.5%	
Randomized	157	26.3%	55	37.9%	102	22.5%	
Study start date							
2015	90	15.1%	12	8.3%	78	17.2%	
2016	74	12.4%	13	9.0%	61	13.5%	
2017	106	17.7%	23	15.9%	83	18.3%	
2018	117	19.6%	30	20.7%	87	19.2%	
2019	121	20.2%	40	27.6%	81	17.9%	
2020	90	15.1%	27	18.6%	63	13.9%	

When comparing studies assessing MRD to those that did not, we observed that there were significant differences in study characteristics (Table 1). There were no studies of supportive care involving MRD assessment (*p* < 0.001). Studies with MRD assessment were more likely to be Phase 2 (50.3% vs. 31.6%, *p* < 0.001) or Phase 3 (22.8% vs. 7.1%, *p* < 0.001) and less likely to be Phase 1 (12.4% vs. 36.2%, *p* < 0.001). Furthermore, we found that studies assessing MRD were more likely aimed at NDMM (33.1% vs. 15.0%, *p* < 0.001) or to involve maintenance therapy (11.0% vs. 5.3%, *p* = 0.02). Studies with MRD assessment also were more likely randomized (37.9% vs. 22.5 %, *p* < 0.001).

Acknowledging the fact that ClinicalTrials.gov does not typically include a full trial protocol, we performed a manual search for published data (article or peer-reviewed abstract) or trial protocols. Of 145 studies that included MRD assessment, such additional data was available for 79 studies (54.4%). Among these 79 studies, 75.9% (*n* = 60) included information on the MRD assessment in either the publication or protocol. Of the 60 studies with information on MRD, NGS-based methods were used in 38.3% (*n* = 23) and flow-based methods were used in 33.3% (*n* = 20) studies, with 4 studies using a combination of both and 21.7% (*n* = 13) of studies not specifying methodology. Among the 60 studies for which information on how MRD was assessed was available in a publication or protocol, a sensitivity of 1/10^5^ was used most often (*n* = 29, 48.3%). The detection threshold was left unspecified in 31.7% (*n* = 19) of studies.

The proportion of trials with MRD assessment showed a clear upward trend from 13.3% in 2015 up to 33.1% in 2019 and 30.0% in 2020 (Fig. 1). A decrease in the fraction of randomized studies with MRD assessment was observed (58.3% in 2015 to 37.0% in 2020). The number of studies that stratified treatment based on MRD showed an increasing trend (0 in 2015 to 3 in 2020).

**Fig. 1:**
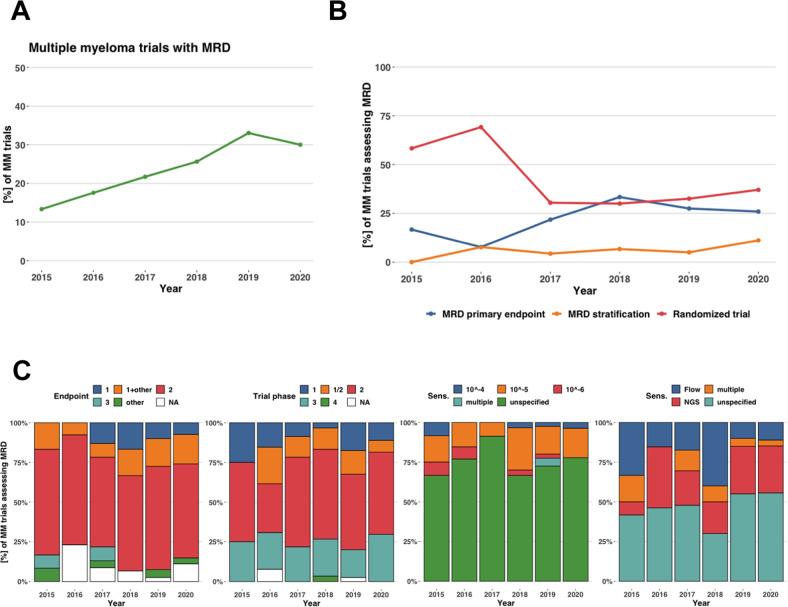
Percentage of multiple myeloma clinical trials with measurable residual disease (MRD) and their characteristics over time as reported within ClinicalTrials.gov. **A** Percentage of all clinical trials for multiple myeloma (MM) that included MRD assessment split by year of clinical trial start date. **B** Within MM trials assessing MRD, the percentage of trials that include MRD as a part of the primary endpoint (blue), that include stratification based on MRD (orange) and that are randomized (red) and the change over time. **C** Within MM trials assessing MRD, distribution of endpoint, trial phase, MRD sensitivity and MRD measurement method and the change over time.

In this comprehensive analysis of MRD assessment in the landscape of myeloma clinical trials over the last five years, we observe that MRD is most commonly measured as an exploratory or secondary endpoint in non-randomized trials. We also demonstrate that it is measured in a heterogeneous fashion with varying methodology/sensitivity across trials and was rarely used to adapt decision making. It must be noted, however, that more recently, several trials have begun to implement MRD in decision making [9, 10] and efforts have been made to harmonize MRD reporting [11]. The current variability in collecting, reporting and analysis limits the immediate clinical applicability of MRD.

Published analyses of studies have often compared survival between those who achieve MRD in both arms of the study to those who do not, in a sense comparing those with responsive disease biology to those with non-responsive disease biology [6, 12]. This is not a valid way to establish trial-level surrogacy, which would be done by demonstrating that between-arm differences in MRD status predict the between-arm differences in survival [13]. Furthermore, the lack of imaging during MRD assessment in many of these trials limits the understanding of disease response, as patients can have residual disease on metabolic imaging even with a complete serological remission and bone marrow MRD negativity [14]. Ongoing technological advances beyond bone marrow NGS/NGF analysis, including, imaging, mass spectrometry and circulating tumor DNA-based methods further complicate harmonization of MRD assessment and require further study. Furthermore, the precise depth of MRD negativity that suffices as a prognostic marker may indeed be different than the depth needed for surrogacy.

Our study has limitations. It relies on data provided by sponsors to ClinicalTrials.gov, and not a review of individual protocols. As such, the high rate of “unspecified” methodology of assessment likely reflects lack of information on ClinicalTrials.gov. Although a manual search of published data was able to provide clarification for some trials, many are still ongoing without results or protocols publicly available yet, limiting our ability to draw inferences. The decision to use ClinicalTrials.gov as a search strategy as opposed to published literature was made, keeping in mind the nascency of the MRD field, with a desire to capture recent studies. However, it must be noted that since MRD is a relatively new field, it is not surprising that it has been captured in such a heterogenous fashion when looking retrospectively at studies listed on ClinicalTrials.gov. Furthermore, although we observed significant heterogeneity in how MRD was assessed, it must be noted that following approval of the Adaptive ClonoSEQ assay by the FDA [15], recent trials (DRAMMATIC, MASTER-2) have used this assay, which may reduce heterogeneity of assessment in the future. Our analysis also does not include recently listed protocols on ClinicalTrials.gov such as NCT04934475/MIDAS and NCT05231629/MASTER-2.

As our understanding and use of MRD matures, it has the potential to help individualize treatment, as is being tested in numerous ongoing randomized trials. However, our analysis of MRD use in clinical trials of MM between 2015 and 2020 demonstrates that it has so far largely been used in an exploratory fashion, and measured in heterogenous ways, limiting its immediate interpretation, applicability, and suitability as a surrogate for overall survival, despite its established use as a prognostic marker. Future studies will need to provide clear guidance around the role of MRD in guiding treatment decisions, which will ultimately increase its applicability beyond clinical trials.

## Supplementary information

Supplementary Figure 1

Supplemental Table 1

Checklist as required at resubmission

## Data Availability

This data was gathered from a publicly available source (ClinicalTrials.gov). The data may be shared upon reasonable request to the corresponding author.

## References

[1] Rajkumar SV (2020). Multiple myeloma: 2020 update on diagnosis, risk-stratification and management. Am J Hematol.

[2] Holstein SA, Suman VJ, McCarthy PL (2019). Should overall survival remain an endpoint for multiple myeloma trials?. Curr Hematol Malig Rep.

[3] Kumar S, Paiva B, Anderson KC, Durie B, Landgren O, Moreau P (2016). International Myeloma Working Group consensus criteria for response and minimal residual disease assessment in multiple myeloma. Lancet Oncol.

[4] Avet-Loiseau H, Ludwig H, Landgren O, Paiva B, Morris C, Yang H (2020). Minimal residual disease status as a surrogate endpoint for progression-free survival in newly diagnosed multiple myeloma studies: a meta-analysis. Clin Lymphoma Myeloma Leuk.

[5] Cavo M, San-Miguel J, Usmani SZ, Weisel K, Dimopoulos MA, Avet-Loiseau H (2022). Prognostic value of minimal residual disease negativity in myeloma: combined analysis of POLLUX, CASTOR, ALCYONE, and MAIA. Blood.

[6] Munshi NC, Avet-Loiseau H, Rawstron AC, Owen RG, Child JA, Thakurta A (2017). Association of minimal residual disease with superior survival outcomes in patients with multiple myeloma: a meta-analysis. JAMA Oncol.

[7] San-Miguel J, Avet-Loiseau H, Paiva B, Kumar S, Dimopoulos MA, Facon T (2022). Sustained minimal residual disease negativity in newly diagnosed multiple myeloma and the impact of daratumumab in MAIA and ALCYONE. Blood.

[8] Wesson W, Galate VL, Sborov DW, McClune B, Goodman AM, Gyawali B (2022). Characteristics of clinical trials for haematological malignancies from 2015 to 2020: A systematic review. Eur J Cancer.

[9] Costa LJ, Chhabra S, Medvedova E, Dholaria BR, Schmidt TM, Godby KN, et al. Daratumumab, Carfilzomib, Lenalidomide, and Dexamethasone With Minimal Residual Disease Response-Adapted Therapy in Newly Diagnosed Multiple Myeloma. J of Clin Oncol. 0:JCO.21.01935. 10.1200/JCO.21.01935 34898239

[10] Krishnan A, Hoering A, Hari P, Sexton R, Orlowski RZ (2020). Phase III Study of Daratumumab/rhuph20 (nsc- 810307) + Lenalidomide or Lenalidomide As Post-Autologous Stem Cell Transplant Maintenance Therapyin Patients with Multiple Myeloma (mm) Using Minimal Residual Disease Todirect Therapy Duration (DRAMMATIC study): SWOG s1803. Blood.

[11] Costa LJ, Derman BA, Bal S, Sidana S, Chhabra S, Silbermann R (2021). International harmonization in performing and reporting minimal residual disease assessment in multiple myeloma trials. Leukemia.

[12] Munshi NC, Avet-Loiseau H, Anderson KC, Neri P, Paiva B, Samur M (2020). A large meta-analysis establishes the role of MRD negativity in long-term survival outcomes in patients with multiple myeloma. Blood Adv.

[13] Little RF, McShane LM, Freidlin B (2017). Myeloma Minimal Residual Disease and Surrogacy. JAMA Oncol.

[14] Rasche L, Alapat D, Kumar M, Gershner G, McDonald J, Wardell CP (2019). Combination of flow cytometry and functional imaging for monitoring of residual disease in myeloma. Leukemia.

[15] FDA authorizes first next generation sequencing-based test to detect very low levels of remaining cancer cells in patients with acute lymphoblastic leukemia or multiple myeloma 2018 [Available from: https://www.fda.gov/news-events/press-announcements/fda-authorizes-first-next-generation-sequencing-based-test-detect-very-low-levels-remaining-cancer.

