# Supplementary material for: Characteristics of measurable residual disease assessment in myeloma: a review of clinical trials from 2015–2020
Source: Blood Cancer J. 2022 Nov 15;12(11):155. doi: 10.1038/s41408-022-00750-1 (PMC9666639; doi:10.1038/s41408-022-00750-1)

Supplementary Figure 1 -- Study selection flowchart.

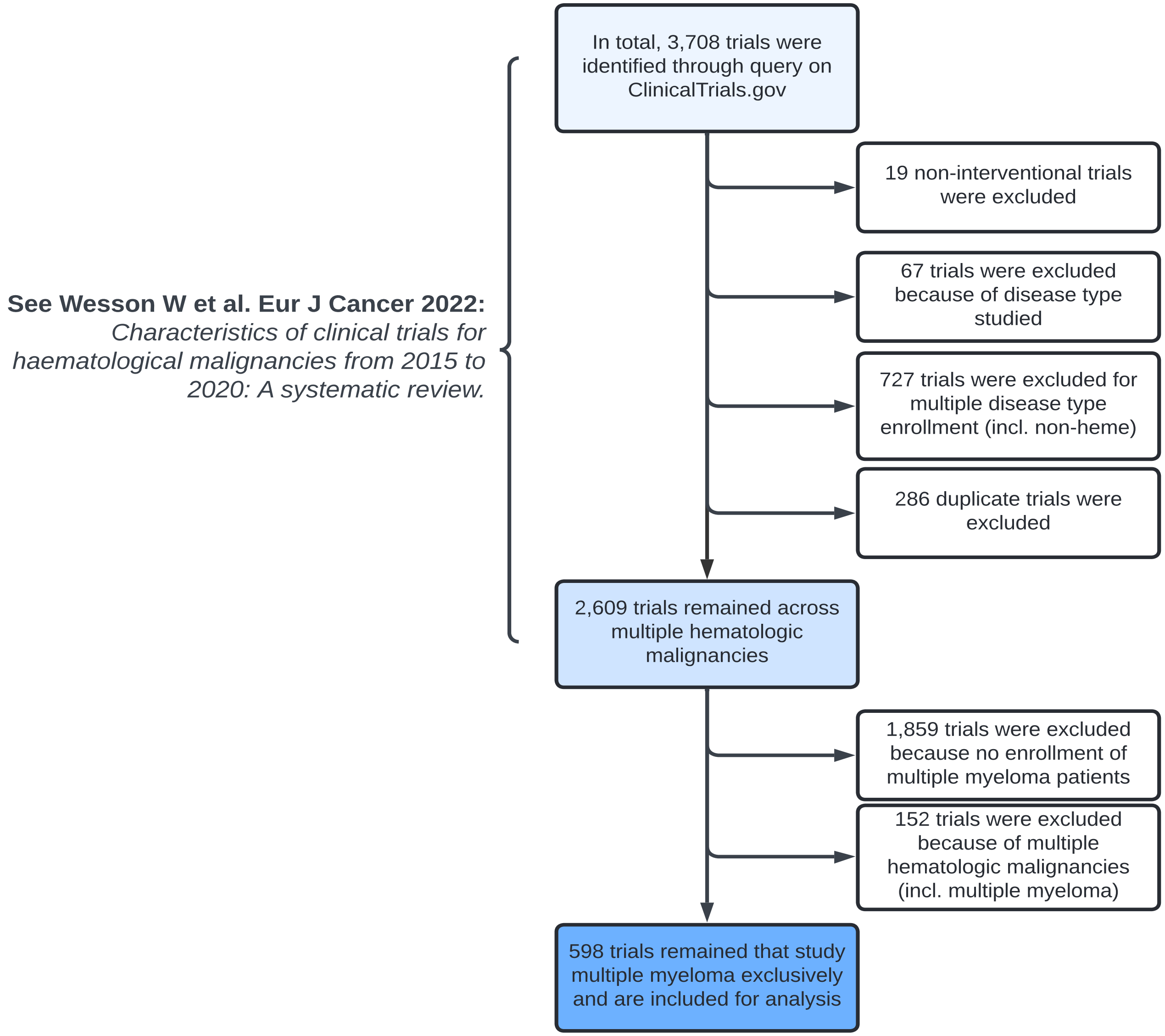

Supplement: Supplementary file 1 — Supplementary Figure 1 [file 41408_2022_750_MOESM1_ESM.pdf]
