# Supplementary material for: Characteristics of measurable residual disease assessment in myeloma: a review of clinical trials from 2015–2020
Source: Blood Cancer J. 2022 Nov 15;12(11):155. doi: 10.1038/s41408-022-00750-1 (PMC9666639; doi:10.1038/s41408-022-00750-1)
Supplement: Supplementary file 2 — Supplemental Table 1 [file 41408_2022_750_MOESM2_ESM.pdf]

	Supplementary Table 1 -- Summarized characteristics of the 598 clinical trials included in the analysis					
**NCT #**	**Name of Intervention**	**Randomization**	**Location of Study**	**Start Year**	**MRD assessment on ClinicalTrials.gov**	**Published**
NCT03975907	CT053	Non-randomized	Non-US	2019	Y	abstract
NCT04151667	Dexamethasone, Bortezomib, Lenalidomide and Daratumumab	Non-randomized	US	2019	Y	not published
NCT02561962	AMG 224	Non-randomized	US	2015	Y	article
NCT03651128	bb2121, Daratumumab, Pomalidomide, Dexamethasone, Bortezomib, Ixazomib, Lenalidomide, Carfilzomib, Elotuzumab	Randomized	Multicenter including US	2018	Y	abstract
NCT03601078	bb2121	Non-randomized	Multicenter including US	2018	Y	not published
NCT04003168	Anti-BCMA CAR-T	Non-randomized	Non-US	2019	Y	not published
NCT03539744	Pomalidomide, Dexamethasone, Venetoclax	Randomized	Multicenter including US	2018	Y	abstract
NCT02899052	Carfilzomib, Venetoclax, Dexamethasone	Non-randomized	Multicenter including US	2017	Y	article
NCT04236011	GC012F	Non-randomized	Non-US	2020	Y	abstract
NCT03393273	Elotuzumab	Non-randomized	Non-US	2018	Y	not published
NCT04194931	Anti-BCMA CAR-T and anti-CD19 CAR-T	Non-randomized	Non-US	2019	Y	not published
NCT02951117	Venetoclax, ABBV-838, Dexamethasone	Non-randomized	Non-US	2017	Y	not published
NCT04162210	Belantamab mafodotin, Pomalidomide, Dexamethasone	Randomized	Multicenter including US	2020	Y	abstract
NCT04617704	GC012F	Non-randomized	Non-US	2020	Y	not published
NCT03785184	Venetoclax, Lenalidomide, Dexamethasone	Non-randomized	Multicenter including US	2019	Y	not published
NCT04484623	Belantamab mafodotin, Pomalidomide, Dexamethasone, Bortezomib	Randomized	Multicenter	2020	Y	abstract

			including US			
NCT03993912	Daratumumab, Lenalidomide, Dexamethasone	Randomized	Non-US	2019	Y	not published
NCT04268498	Daratumumab, Lenalidomide, Carfilzomib, Dexamethasone	Randomized	Multicenter including US	2020	Y	not published
NCT03815279	Carfilzomib, Lenalidomide, Dexamethasone	Non-randomized	Non-US	2019	Y	not published
NCT03361748	bb2121	Non-randomized	Multicenter including US	2017	Y	article
NCT03669445	Ixazomib, Lenalidomide, Dexamethasone, Daratumumab	Non-randomized	Non-US	2018	Y	abstract
NCT03224507	Carfilzomib, Lenalidomide, Dexamethasone, Daratumumab, Autologous Stem Cell Transplant	Non-randomized	US	2018	Y	article
NCT02969837	Elotuzumab, Carfilzomib, Lenalidomide, Dexamethasone	Non-randomized	US	2017	Y	abstract
NCT02655458	Elotuzumab, Lenalidomide, Autologous Stem Cell Transplant	Non-randomized	US	2016	Y	abstract
NCT03314181	Daratumumab, Venetoclax, Bortezomib, Dexamethasone	Randomized	Multicenter including US	2018	Y	article
NCT02659293	Lenalidomide, Carfilzomib, Dexamethasone	Randomized	Multicenter including US	2016	Y	not published
NCT04246047	Belantamab Mafodotin, Daratumumab, Bortezomib, Dexamethasone	Randomized	Multicenter including US	2020	Y	abstract
NCT03376477	GM-CSF Vaccine, Prevnar13, Lenalidomide, Placebo	Randomized	US	2019	Y	article
NCT02728102	Dendritic Cell/Myeloma Fusion Vaccine, Lenalidomide, GM-CSF	Randomized	US	2016	Y	abstract
NCT04288765	Carfilzomib, Daratumumab, Lenalidomide, Dexamethasone	Non-randomized	Non-US	2020	Y	not published
NCT03606577	Carfilzomib, Daratumumab, Lenalidomide, Dexamethasone, Tandem Autologous Stem Cell Transplantation	Non-randomized	Non-US	2019	Y	not published
NCT04661137	Selinexor, Carfilzomib, Daratumumab, Pomalidomide	Non-randomized	US	2020	Y	not published
NCT04280328	Ciforadenant, Daratumumab	Non-randomized	US	2020	Y	not published
NCT04108624	Discontinuation of Lenalidomide, Pomalidomide, Bortezomib, Ixazomib	Non-randomized	US	2019	Y	not published

NCT02720510	Lenalidomide, Bortezomib, Dexamethasone, Panobinostat	Randomized	US	2016	Y	not published
NCT04140162	Daratumumab, Lenalidomide, Bortezomib, Dexamethasone	Non-randomized	US	2020	Y	not published
NCT04483739	Isatuximab, Carfilzomib, Lenalidomide, Dexamethasone	Randomized	Non-US	2020	Y	not published
NCT02252172	Daratumumab, Lenalidomide, Dexamethasone	Randomized	Multicenter including US	2015	Y	article
NCT02253316	Ixazomib, Lenalidomide, Dexamethasone	Randomized	US	2015	Y	abstract
NCT03761108	REGN5458	Non-randomized	Multicenter including US	2019	Y	abstract
NCT04133636	BCMA CAR-T, Lenalidomide, Daratumumab, Bortezomib, Dexamethasone	Non-randomized	Multicenter including US	2019	Y	abstract
NCT02891811	Carfilzomib, Thalidomide, Lenalidomide, Dexamethasone	Randomized	Non-US	2017	Y	not published
NCT03837509	INCB001158, Daratumumab	Randomized	Multicenter including US	2019	Y	not published
NCT02685826	Durvalumab, Leanlidomide, Dexamethasone	Randomized	Multicenter including US	2016	Y	abstract
NCT03290950	Daratumumab, Carfilzomib, Lenalidomide, Dexamethasone	Non-randomized	US	2017	Y	abstract
NCT02703779	Bortezomib, G-CSF, Plerixafor	Randomized	US	2016	Y	abstract
NCT03412565	Daratumumab, Bortezomib, Lenalidomide, Dexamethasone, Melphalan, Prednisone, Carfilzomib	Non-randomized	Multicenter including US	2018	Y	article
NCT03875495	Temferon	Non-randomized	Non-US	2019	Y	not published
NCT03288493	P-BCMA-101 CAR-T	Non-randomized	US	2017	Y	abstract
NCT03871829	Daratumumab, Carfilzomib, Dexamethasone	Randomized	Multicenter including US	2019	Y	abstract
NCT03729804	Carfilzomib, Lenalidomide, Dexamethasone, Bortezomib	Randomized	US	2019	Y	not published
NCT03746652	Daratumumab, Ixazomib, Dexamethasone	Non-randomized	Non-US	2018	Y	abstract

NCT03758417	LCAR-B28M CAR-T	Non-randomized	Non-US	2019	Y	not published
NCT03710603	Daratumumab, Bortezomib, Lenalidomide, Dexamethasone	Randomized	Non-US	2018	Y	abstract
NCT03700450	Cyclophosphamide	Non-randomized	Non-US	2018	Y	article
NCT03548207	JNJ-68284528	Non-randomized	Multicenter including US	2018	Y	article
NCT04181827	JNJ-68284528, Pomalidomide, Bortezomib, Dexamethasone, Daratumumab	Randomized	Multicenter including US	2020	Y	not published
NCT02756728	BI-505, Melphalan, Autologous Stem Cell Transplant	Randomized	US	2016	Y	not published
NCT02780609	Selinexor, Melphalan, Dexamethasone, Fosaprepitant, Autologous Stem Cell Transplantation	Non-randomized	US	2017	Y	abstract
NCT02755597	Bortezomib, Dexamethasone, Venetoclax, Placebo	Randomized	Multicenter including US	2016	Y	article
NCT03652064	Daratumumab, Bortezomib, Lenalidomide, Dexamethasone	Randomized	Multicenter including US	2018	Y	abstract
NCT03180736	Daratumumab, Pomalidomide, Dexamethasone	Randomized	Non-US	2017	Y	article
NCT02874742	Lenalidomide, Bortezomib, Dexamethasone, Daratumumab	Randomized	US	2017	Y	article
NCT04083534	REGN5459	Non-randomized	US	2019	Y	not published
NCT02312258	Ixazomib, Placebo	Randomized	Multicenter including US	2015	Y	article
NCT03168100	Elotuzumab, Lenalidomide, Dexamethasone, Bortezomib	Non-randomized	US	2017	Y	not published
NCT02514239	BI 836909	Non-randomized	Non-US	2015	Y	article
NCT04287855	Isatuximab, Carfilzomib, Pomalidomide	Non-randomized	Non-US	2020	Y	not published
NCT03860038	TJ202, Dexamethasone	Non-randomized	Non-US	2019	Y	not published
NCT04124497	Daratumumab, Pomalidomide, Dexamethasone	Non-randomized	Non-US	2019	Y	not published
NCT03155100	Carfilzomib, Elotuzumab, Dexamethasone	Non-randomized	Non-US	2017	Y	abstract
NCT03590652	Ixazomib, Pomalidomide, Dexamethasone, Daratumumab	Non-randomized	US	2018	Y	abstract

NCT03697655	Daratumumab	Randomized	Non-US	2018	Y	abstract
NCT03158688	Carfilzomib, Daratumumab, Dexamethasone	Randomized	Multicenter including US	2017	Y	article
NCT03030261	Elotuzumab, Pomalidomide, Dexamethasone	Non-randomized	Multicenter including US	2017	Y	not published
NCT03490344	Daratumumab, Lenalidomide	Non-randomized	US	2018	Y	not published
NCT03500445	Daratumumab, Carfilzomib, Lenalidomide, Dexamethasone	Non-randomized	US	2019	Y	not published
NCT03901963	Daratumumab, Lenalidomide	Randomized	US	2019	Y	abstract
NCT03720041	Ixazomib, Lenalidomide, Dexamethasone, Placebo	Randomized	Non-US	2019	Y	abstract
NCT03287908	AMG 701, Pomalidomide, Dexamethasone	Non-randomized	Multicenter including US	2017	Y	abstract
NCT03944057	Selinexor, Dexamethasone	Non-randomized	Non-US	2019	Y	article
NCT02937571	Carfilzomib, Dexamethasone, Lenalidomide	Non-randomized	US	2016	Y	not published
NCT03562169	Ixazomib, Thalidomide, Dexamethasone, Conventional autologous stem cell transplant, augmented autologous stem cell transplant	Randomized	Non-US	2017	Y	article
NCT03617731	Lenalidomide, Bortezomib, Dexamethasone, Isatuximab	Randomized	Non-US	2018	Y	abstract
NCT04093596	ALLO-715, ALLO-647, Fludarabine, Cyclophosphamide, Nirogacestat	Non-randomized	US	2019	Y	abstract
NCT03992170	Daratumumab	Non-randomized	Non-US	2018	Y	abstract
NCT03859427	Carfilzomib, Dexamethasone, Lenalidomide	Randomized	Multicenter including US	2019	Y	abstract
NCT04309981	BCMA CAR-T	Non-randomized	Non-US	2020	Y	abstract
NCT04430894	Carfilzomib, Isatuximab, Lenalidomide, Dexamethasone	Non-randomized	US	2020	Y	abstract
NCT03473496	BCMA/CD138/CD38/CD56 CAR-T	Non-randomized	Non-US	2018	Y	not published
NCT03836053	AMG 420	Non-randomized	Multicenter including US	2019	Y	not published
NCT02203643	Carfilzomib, Cyclophosphamide, Lenalidomide, Dexamethasone	Randomized	Non-US	2015	Y	article

NCT04467281	89Zr-DFO-Daratumumab PET/CT	Non-randomized	US	2020	Y	not published
NCT03416374	Ixazomib, Lenalidomide, Bortezomib, Carfilzomib, Dexamethasone	Non-randomized	Non-US	2018	Y	not published
NCT02315716	Carfilzomib, Cyclophosphamide, Dexamethasone, Autologous Stem Cell Transplant	Randomized	Non-US	2015	Y	abstract
NCT03650491	FOR46	Non-randomized	US	2019	Y	abstract
NCT03376672	Ixazomib, Lenalidomide, Dexamethasone	Non-randomized	Non-US	2018	Y	abstract
NCT03792620	Daratumumab, Cyclophosphamide, Thalidomide, Dexamethasone	Non-randomized	Non-US	2018	Y	abstract
NCT04436029	Descartes 11	Non-randomized	US	2020	Y	not published
NCT03910439	Avelumab, External Beam Radiotherapy	Non-randomized	US	2019	Y	not published
NCT02981199	Microtransplantation, Autologous Stem Cell Transplant	Randomized	Non-US	2016	Y	not published
NCT03413800	Lenalidomide, Dexamethasone, Donor Lymphocyte Infusion	Non-randomized	Non-US	2018	Y	not published
NCT04508790	Leflunomide, Pomalidomide, Dexamethasone	Non-randomized	US	2020	Y	not published
NCT04096066	Carfilzomib, Lenalidomide, Dexamethasone	Randomized	Non-US	2019	Y	not published
NCT03942224	Bortezomib, Daratumumab, Dexamethasone, Ixazomib	Randomized	US	2019	Y	not published
NCT03841565	Daratumumab, Pomalidomide, Dexamethasone	Non-randomized	US	2020	Y	not published
NCT03012880	Daratumumab, Ixazomib, Lenalidomide, Dexamethasone	Non-randomized	US	2017	Y	abstract
NCT03701321	Daratumumab, Bortezomib, Dexamethasone, Venetoclax	Non-randomized	US	2019	Y	abstract
NCT04205240	Daratumumab, Cyclophosphamide, Fludarabine, Melphalan, Mycophenolate Mofetil, Tacrolimus, Allogeneic Stem Cell Transplant	Non-randomized	US	2020	Y	not published
NCT03417284	Melphalan Hydrochloride, Filgrastim-sndz, Autologous Stem Cell Transplant	Randomized	US	2019	Y	not published
NCT03275285	Isatuximab, Carfilzomib, Dexamethasone	Randomized	Multicenter including US	2017	Y	article
NCT03202628	Ixazomib, Pomalidomide, Dexamethasone, Autologous Stem Cell Transplant	Non-randomized	US	2017	Y	not published

NCT02990338	Isatuximab, Pomalidomide, Dexamethasone	Randomized	Multicenter including US	2016	Y	article
NCT02513186	Isatuximab, Bortezomib, Cyclophosphamide, Dexamethasone, Lenalidomide	Non-randomized	Non-US	2015	Y	abstract
NCT04071457	Lenalidomide, Daratumumab, rHuPH20	Randomized	US	2019	Y	abstract
NCT03896737	Daratumumab, Bortezomib, Cyclophosphamide, Thalidomide, Dexamethasone	Randomized	Non-US	2019	Y	not published
NCT03104842	Isatuximab, Carfilzomib, Lenalidomide, Dexamethasone	Non-randomized	Non-US	2017	Y	article
NCT02955810	Daratumumab, Cyclophosphamide, Bortezomib, Dexamethasone	Non-randomized	Non-US	2016	Y	article
NCT02636010	Pembrolizumab	Non-randomized	Non-US	2016	Y	article
NCT03188172	Cyclophosphamide, Bortezomib, Lenalidomide, Daratumumab, Dexamethasone, Melphalan, Filgrastim	Non-randomized	Non-US	2017	Y	abstract
NCT04566328	Bortezomib, Daratumumab, Hyaluronidase-fihj, Dexamethasone, Lenalidomide	Randomized	US	2020	Y	abstract
NCT03756896	Carfilzomib, Dexamethasone, Pomalidomide	Non-randomized	US	2019	Y	not published
NCT02575144	Clarithromycin, Lenalidomide, Dexamethasone	Randomized	Non-US	2015	Y	abstract
NCT04191616	Carfilzomib, Dexamethasone, Pomalidomide	Non-randomized	Multicenter including US	2020	Y	abstract
NCT03763162	Bortezomib, Daratumumab, Dexamethasone, Ixazomib	Non-randomized	US	2019	Y	not published
NCT03314636	Carfilzomib, Lenalidomide, Dexamethasone, PET-CT	Non-randomized	Non-US	2018	Y	abstract
NCT03948035	Elotuzumab, Carfilzomib, Lenalidomide, Dexamethasone, Autologous Stem Cell Transplant	Randomized	Non-US	2018	Y	not published
NCT03477539	Daratumumab, Lenalidomide, Autologous Stem Cell Transplant	Non-randomized	US	2018	Y	not published
NCT03346135	Daratumumab, Melphalan, Autologous Stem Cell Transplant	Non-randomized	US	2019	Y	not published
NCT02389517	Ixazomib Citrate, Lenalidomide, Dexamethasone	Randomized	US	2015	Y	not published
NCT04513639	Carfilzomib, Dexamethasone, Daratumumab	Randomized	Non-US	2020	Y	abstract
NCT03687125	Tinostamustine	Non-randomized	Multicenter including US	2018	Y	not published

NCT03602235	High Dose Abscorbic Acid, Melphalan	Non-randomized	US	2019	Y	not published
NCT03100877	Autologous Stem Cell Transplant, Cyclophosphamide, Filgrastim, Lenalidomide, Melphalan, Palifermin, Total Marrow Radiation	Non-randomized	US	2017	Y	not published
NCT04009109	Lenalidomide, Ixazomib, Daratumumab, Dexamethasone	Randomized	US	2020	Y	abstract
NCT04176718	Daratumumab, Carfilzomib, Pomalidomide, Dexamethasone	Non-randomized	US	2020	Y	not published
NCT03319667	Isatuximab, Bortezomib, Lenalidomide, Dexamethasone, Acetaminophen, Ranitidine, Diphenhydramine	Randomized	Multicenter including US	2017	Y	abstract
NCT03455972	anti-CD19 CAR T, anti-BCMA CAR-T	Non-randomized	Non-US	2018	Y	article
NCT02547662	Ixazomib, Pomalidomide, Dexamethasone	Non-randomized	US	2015	Y	abstract
NCT04557098	Teclistamab	Non-randomized	Multicenter including US	2020	Y	article
NCT02506959	Busulfan, Gemcitabine, Melphalan, Panobinostat, Autologous Stem Cell Transplant	Non-randomized	US	2015	Y	not published
NCT03303950	Busulfan, Cyclophosphamide, Fludarabine, Hematopoietic Cel Transplant	Non-randomized	US	2018	Y	not published
NCT04309084	CYNK-001	Randomized	US	2020	Y	not published
NCT04174196	CC-486, Lenalidomide	Non-randomized	US	2019	Y	abstract
NCT03328936	Melphalan Hydrochloride	Randomized	US	2018	Y	not published
NCT04322292	C-CAR088	Non-randomized	Non-US	2019	N	NA
NCT04295018	C-CAR088	Non-randomized	Non-US	2019	N	NA
NCT04537442	IM21	Non-randomized	Non-US	2020	N	NA
NCT03670173	Osalmid	Non-randomized	Non-US	2018	N	NA
NCT03757221	Ixazomib and Daratumumab	Non-randomized	Non-US	2019	N	NA
NCT03782064	Dendritic Cell/MM Fusion vaccine and Nivolumab	Non-randomized	US	2019	N	NA
NCT03657420	ABI-009 and Pomalidomide and Dexamethasone	Non-randomized	US	2019	N	NA
NCT04318327	PHE885	Non-randomized	US	2020	N	NA
NCT03091257	Dabrafenib and/or Trametinib	Non-randomized	US	2017	N	NA

NCT03227432	Elotuzumab and Nivolumab and Pomalidomide	Non-randomized	US	2018	N	NA
NCT03379584	SGN-CD48A	Non-randomized	US	2018	N	NA
NCT03104270	Elotuzumab and Pomalidomide and Carfilzomib and Dexamethasone	Non-randomized	US	2017	N	NA
NCT02477215	Bendamustin and Ixazomib and Dexamethasone	Non-randomized	US	2015	N	NA
NCT03848845	GSK2857916 and Pembrolizumab	Non-randomized	Multicenter including US	2019	N	NA
NCT03492138	Ixazomib and ONC201 and Dexamethasone	Non-randomized	US	2018	N	NA
NCT02441686	Lenalidomide and Bortezomib and Dexamethasone	Non-randomized	US	2015	N	NA
NCT02963493	Melphalan Flufenamide and Dexamethasone	Non-randomized	Multicenter including US	2016	N	NA
NCT02402725	Dexamethasone	Non-randomized	US	2015	N	NA
NCT04674813	CC-95266	Non-randomized	US	2020	N	NA
NCT04123418	WVT078 and WHG626	Non-randomized	Multicenter including US	2019	N	NA
NCT04024384	Daratumumab	Non-randomized	Non-US	2019	N	NA
NCT02718833	Elotuzumab and Pomalidomide and Bortezomib and Dexamethasone	Non-randomized	US	2016	N	NA
NCT04398485	ION251	Non-randomized	US	2020	N	NA
NCT04309942	Guided Pharmacy Consultation	Randomized	Non-US	2020	N	NA
NCT02416206	Bendamustine, Etoposide, Cytarabine, Melphalan	Non-randomized	US	2015	N	NA
NCT03549442	CART-BCMA and huCART19	Randomized	US	2018	N	NA
NCT03309111	ISB 1342 (BiTE)	Non-randomized	US	2017	N	NA
NCT02833610	Denosumab	Non-randomized	US	2016	N	NA
NCT03767725	Anti-BCMA CART and Anti-CD19 CART	Non-randomized	Non-US	2018	N	NA
NCT02802163	Carfilzomib, Lenalidomide, Dexamethasone, Panobinostat	Non-randomized	US	2017	N	NA
NCT02541383	Bortezomib, Thalidomide, Dexamethasone, Daratumumab	Randomized	Non-US	2015	N	NA
NCT03287804	AUTO2	Non-randomized	Non-US	2017	N	NA
NCT03915184	CT053	Non-randomized	US	2019	N	NA
NCT02462525	ABBV-838, Pomalidomide, Dexamethasone	Non-randomized	Multicenter	2015	N	NA

			including US			
NCT03989414	CC-92480, Bortezomib, Dexamethasone, Daratumumab, Carfilzomib	Randomized	Multicenter including US	2019	N	NA
NCT04541368	CS-1 Targeted CAR T	Non-randomized	Non-US	2020	N	NA
NCT02967276	Metformin and Dexamethasone	Non-randomized	Non-US	2017	N	NA
NCT03292263	Nivolumab with ASCT	Non-randomized	Non-US	2017	N	NA
NCT04244656	CTX120	Non-randomized	Multicenter including US	2020	N	NA
NCT03943472	anti-BCMA CAR-T and Immune Inhibitors	Randomized	Non-US	2019	N	NA
NCT03117361	Plitidepsin, Bortezomib, Dexamethasone	Non-randomized	Non-US	2017	N	NA
NCT04519476	Selinexor, Lenalidomide, Methylprednisolone	Non-randomized	US	2020	N	NA
NCT04243109	Pomalidomide, Cyclophosphamide, Dexamethasone	Non-randomized	Non-US	2017	N	NA
NCT03033316	Oncocort	Non-randomized	Non-US	2017	N	NA
NCT03994705	Descartes-11	Non-randomized	US	2019	N	NA
NCT02669615	Melphalan HCl	Non-randomized	US	2016	N	NA
NCT02573935	Clarithromycin, Bortezomib, Cyclophosphamide, Dexamethasone, Placebo	Randomized	Non-US	2015	N	NA
NCT02220608	Bortezomib, G-CSF	Non-randomized	US	2015	N	NA
NCT03000452	Daratumumab, Durvalumab	Non-randomized	Multicenter including US	2017	N	NA
NCT02576977	Pembrolizumab, Pomalidomide, Dexamethasone	Randomized	Multicenter including US	2015	N	NA
NCT04036461	CC-99712	Non-randomized	Multicenter including US	2019	N	NA
NCT02939183	Oprozomib, Pomalidomide, Dexamethasone	Non-randomized	Multicenter including US	2017	N	NA
NCT02519452	Daratumumab, Recombinant Human Hyaluronidase	Non-randomized	Multicenter including US	2015	N	NA
NCT02658929	bb2121	Non-randomized	US	2015	N	NA

NCT04196491	bb2121, Lenalidomide	Non-randomized	US	2020	N	NA
NCT04394650	CC-98633	Non-randomized	US	2020	N	NA
NCT02519114	Haploidentical transplant	Non-randomized	Non-US	2015	N	NA
NCT03110562	Selinexor, Bortezomib, Dexamethasone	Randomized	Multicenter including US	2017	N	NA
NCT04570631	Eftozanermin Alfa, Bortezomib, Dexamethasone	Non-randomized	Multicenter including US	2020	N	NA
NCT03110822	Ruxolitinib, Lenalidomide, Methylprednisolone	Non-randomized	US	2017	N	NA
NCT02928029	Xofigo, Placebo, Bortezomib, Dexamethasone	Randomized	Multicenter including US	2017	N	NA
NCT03795597	Carfilzomib, Busulfan, Melphalan	Non-randomized	US	2019	N	NA
NCT04484714	Virtual Exercise	Non-randomized	Non-US	2020	N	NA
NCT03486067	CC-93269	Non-randomized	Multicenter including US	2018	N	NA
NCT03271632	Multi-CAR T	Non-randomized	Non-US	2017	N	NA
NCT02626481	Daratumumab, Dexamethasone	Non-randomized	Non-US	2015	N	NA
NCT04162119	BCMA-PD1-CART	Non-randomized	Non-US	2019	N	NA
NCT04432818	Digital Life Coaching	Non-randomized	US	2020	N	NA
NCT02811822	GMI-1271	Non-randomized	Non-US	2016	N	NA
NCT02278315	I-131-CLR1404, Dexamethasone	Non-randomized	US	2015	N	NA
NCT04119336	Nivolumab, Ixazomib, Dexamethasone, Cyclophosphamide	Non-randomized	US	2020	N	NA
NCT03111992	CJM112, PDR001, LCL161	Non-randomized	Multicenter including US	2017	N	NA
NCT03582033	SEA-BCMA, Dexamethasone	Non-randomized	US	2018	N	NA
NCT03828292	Belantamab mafodotin, Bortezomib, Pomalidomide, Dexamethasone	Non-randomized	Non-US	2019	N	NA
NCT02384083	Filanesib, Pomalidomide, Dexamethasone	Non-randomized	Non-US	2015	N	NA
NCT03525678	Belantamab mafodotin	Randomized	Multicenter including US	2018	N	NA

NCT02605356	Xofigo, Bortezomib, Dexamethasone, Placebo	Randomized	Multicenter including US	2016	N	NA
NCT02291848	TAA-Specific CTL	Non-randomized	US	2015	N	NA
NCT02732925	Balloon Kyphoplasty, Conservative Management	Randomized	Non-US	2017	N	NA
NCT04091126	Belantamab Mafodotin, Bortezomib, Lenalidomide, Dexamethasone	Randomized	Multicenter including US	2019	N	NA
NCT04637269	anti-BCMA CAR-T	Non-randomized	Non-US	2020	N	NA
NCT02646085	C11 Methionine PET	Non-randomized	US	2016	N	NA
NCT02440464	Fludarabine, Melphalan, Bortezomib, Ixazomib, Placebo, Allogeneic Stem Cell Transplant	Randomized	US	2015	N	NA
NCT03567616	Venetoclax, Pomalidomide, Dexamethasone	Non-randomized	Multicenter including US	2018	N	NA
NCT03804424	64Cu-LLP2A PET	Non-randomized	US	2018	N	NA
NCT02851056	Survivin Vaccine and Autologous Stem Cell Transplant	Non-randomized	US	2016	N	NA
NCT04671251	AEVI-007	Non-randomized	US	2020	N	NA
NCT04176380	RAPA-201 T Cells	Non-randomized	US	2020	N	NA
NCT02362165	Bortezomib, Doxorubicin, Dexamethasone, Cyclophosphamide	Randomized	Non-US	2015	N	NA
NCT03430011	JCARH125, Anakinra	Non-randomized	US	2018	N	NA
NCT03269136	PF-06863135, PF-06801591, Lenalidomide, Pomalidomide	Non-randomized	Multicenter including US	2017	N	NA
NCT04493411	Whole Body MRI	Non-randomized	US	2020	N	NA
NCT04126200	Belantamab Mafodotin, GSK3174998, Feladilimab, Nirogacestat, Dostarlimab, Isatuximab	Randomized	Multicenter including US	2019	N	NA
NCT03374085	CC-92480, Dexamethasone	Non-randomized	Multicenter including US	2018	N	NA
NCT02572492	Carfilzomib, Cyclophosphamide, Dexamethasone, Melphalan, Autologous Stem Cell Transplant	Randomized	Non-US	2015	N	NA
NCT04447573	BCMA CAR-T	Non-randomized	Non-US	2020	N	NA
NCT04083898	Isatuximab, Bendamustine, Prednisone	Non-randomized	US	2020	N	NA

NCT04349358	FCH PET, FDG PET	Non-randomized	Non-US	2020	N	NA
NCT03520985	Alternate Day Dosing of Pomalidomide	Non-randomized	Non-US	2018	N	NA
NCT04376957	Oral Chemotherapy Teaching Tool	Non-randomized	Non-US	2020	N	NA
NCT04414475	Selinexor, Dexamethasone, Bortezomib	Randomized	Non-US	2020	N	NA
NCT02628704	Selinexor, Carfilzomib, Dexamethasone, Placebo	Randomized	US	2015	N	NA
NCT02419118	Daratumumab, Lenalidomide, Dexamethasone	Randomized	Non-US	2015	N	NA
NCT03695744	Daratumumab, Bortezomib, Dexamethasone	Non-randomized	Non-US	2019	N	NA
NCT04555551	MCARH109	Non-randomized	US	2020	N	NA
NCT02331368	Autologous Stem Cell Transplant, Melphalan, Lenalidomide, MK-3475	Non-randomized	US	2015	N	NA
NCT04248244	Early Palliative Care	Non-randomized	US	2020	N	NA
NCT02497378	Daratumumab, Bortezomib, Dexamethasone	Non-randomized	Non-US	2015	N	NA
NCT03759093	CURATE.AE -Guided dosage modulation, Bortezomib, Cyclophosphamide, Dexamethasone, Thalidomide	Randomized	Non-US	2020	N	NA
NCT04497961	Lenalidomide, Daratumumab	Randomized	US	2020	N	NA
NCT04499339	SLAMF7 CAR-T	Non-randomized	Non-US	2020	N	NA
NCT03931421	BCMA CAR-T	Non-randomized	Non-US	2019	N	NA
NCT02548962	Ibrutinib, Pomalidomide, Dexamethasone, Placebo	Non-randomized	Multicenter including US	2016	N	NA
NCT03322735	BCMA CAR-T	Non-randomized	Non-US	2017	N	NA
NCT02955550	rhIL-2, PNK-007	Non-randomized	US	2017	N	NA
NCT04552743	MGTA-145, Plerixafor	Non-randomized	US	2020	N	NA
NCT04586426	Talquetamab, Teclistamab	Non-randomized	Non-US	2020	N	NA
NCT03274219	bb21217	Non-randomized	US	2017	N	NA
NCT03042793	PD-L1 Vaccine	Non-randomized	Non-US	2017	N	NA
NCT04561492	[68Ga]Ga-PentixaFor PET	Non-randomized	Non-US	2020	N	NA
NCT04121260	Daratumumab	Non-randomized	Non-US	2019	N	NA
NCT04156269	BCMA-CS1 cCAR T	Non-randomized	Non-US	2018	N	NA
NCT04445701	AO-176, Dexamethasone, Bortezomib	Non-randomized	US	2020	N	NA
NCT04557150	RO7425781	Non-randomized	Multicenter	2020	N	NA

			including US			
NCT04182581	BCMA/CD19 Dual-Target CAR-T	Non-randomized	Non-US	2019	N	NA
NCT02773030	CC-220, Dexamethasone, Daratumumab, Bortezomib, Carfilzomib	Randomized	Multicenter including US	2016	N	NA
NCT03909412	Carfilzomib, Cyclophosphamide, Dexamethasone, GM-CSF	Randomized	US	2019	N	NA
NCT04113018	Daratumumab, Carfilzomib, Lenalidomide, Dexamethasone	Non-randomized	US	2019	N	NA
NCT03933735	TNB-383B	Non-randomized	US	2019	N	NA
NCT02654990	Panbinostat, Bortezomib, Dexamethasone	Randomized	Multicenter including US	2016	N	NA
NCT03489525	MEDI2228	Non-randomized	Multicenter including US	2018	N	NA
NCT02343042	Selinexor, Dexamethasone, Leanlidomide, Pomalidomide, Bortezomib, Daratumumab, Carfilzomib, Ixazomib, Elotuzumab, Clarithromycin, Belantamab Mafodotin	Randomized	Multicenter including US	2015	N	NA
NCT03777306	Myeloma Pack Intervention	Randomized	US	2017	N	NA
NCT02863991	ONC201	Non-randomized	US	2016	N	NA
NCT03665155	89Zr-DFO-daratumumab PET	Non-randomized	US	2018	N	NA
NCT02254551	LDE225, Bortezomib	Non-randomized	US	2015	N	NA
NCT04186052	BMA CAR-T	Non-randomized	Non-US	2019	N	NA
NCT02756663	Panobinostat, Carfilzomib, Dexamethasone	Non-randomized	Multicenter including US	2016	N	NA
NCT03711864	IM21 CAR-T	Non-randomized	Non-US	2019	N	NA
NCT02807454	Daratumumab, Durvalumab, Pomalidomide, Dexamethasone	Randomized	Multicenter including US	2016	N	NA
NCT03773107	Carfilzomib, Ruxolitinib, Dexamethasone	Non-randomized	US	2019	N	NA
NCT04287660	Clarithromycin, Lenalidomide, Dexamethasone, BCMA CAR-T	Non-randomized	Non-US	2017	N	NA
NCT04108195	Daratumumab, Talquetamab, Teclistamab, Pomalidomide	Non-randomized	Multicenter including US	2020	N	NA
NCT04045795	Isatuximab, Pomalidomide, Dexamethasone	Non-randomized	Multicenter	2019	N	NA

			including US			
NCT03767751	CD28 and BCMA CAR-T	Non-randomized	Non-US	2018	N	NA
NCT03544281	Belantamab Mafodotin, Lenalidomide, Dexamethasone, Bortezomib	Non-randomized	Multicenter including US	2018	N	NA
NCT02389543	Selinexor, Lenalidomide, Dexamethasone	Randomized	US	2015	N	NA
NCT04025450	Chidamide, Bortezomib, Lenalidomide, Dexamethasone	Randomized	Non-US	2019	N	NA
NCT03090659	LCAR-B38M CAR-T	Non-randomized	Non-US	2015	N	NA
NCT03262389	F-18 FDG PET, C-11 Acetate PET	Non-randomized	US	2017	N	NA
NCT03288480	PT-112	Non-randomized	US	2017	N	NA
NCT03439293	Ixazomib, Daratumumab, Dexamethasone	Non-randomized	Multicenter including US	2018	N	NA
NCT03093168	BCMA CAR-T	Non-randomized	Non-US	2017	N	NA
NCT03336073	Carfilzomib, Cyclophosphamide, Dexamethasone	Randomized	Non-US	2017	N	NA
NCT03340883	BION-1301	Non-randomized	US	2017	N	NA
NCT03445663	AMG 424	Non-randomized	Multicenter including US	2018	N	NA
NCT03441958	ECT-001 Expanded Cord Blood Transplant	Non-randomized	Non-US	2018	N	NA
NCT02954796	SGN-CD352A	Non-randomized	US	2016	N	NA
NCT03508765	rsfMRI, Neurocognitive testing	Non-randomized	US	2018	N	NA
NCT03984097	TAK-079, Lenalidomide, Dexamethasone, Bortezomib	Non-randomized	US	2019	N	NA
NCT03046329	Carevive CPS	Non-randomized	US	2015	N	NA
NCT02794246	CART-19 Cells	Non-randomized	US	2016	N	NA
NCT03733717	Isatuximab	Non-randomized	Non-US	2018	N	NA
NCT02546167	BCMA CAR-T	Randomized	US	2015	N	NA
NCT03829371	Bortezomib, Melphalan, Prednisone, Lenalidomide, Dexamethasone	Randomized	Non-US	2019	N	NA
NCT03218163	MEDI-551	Non-randomized	US	2017	N	NA
NCT02616640	Durvalumab, Pomalidomide, Dexamethasone	Randomized	Multicenter including US	2016	N	NA

NCT03143985	Vactosertib, Pomalidomide	Non-randomized	US	2017	N	NA
NCT03748953	Ixazomib	Randomized	Non-US	2019	N	NA
NCT03015922	Lenalidomide, Pomalidomide, Reolysin	Non-randomized	Non-US	2017	N	NA
NCT02406222	Pomalidomide, Dexamethasone, Cyclophosphamide	Randomized	Non-US	2016	N	NA
NCT03275103	BFCR4350A, Tocilizumab	Non-randomized	Multicenter including US	2017	N	NA
NCT04177823	Belantamab Mafodotin	Non-randomized	Non-US	2019	N	NA
NCT04155749	CART-ddBCMA	Non-randomized	US	2019	N	NA
NCT02439112	Individualized exercise	Non-randomized	Non-US	2015	N	NA
NCT03242889	Daratumumab	Non-randomized	Non-US	2017	N	NA
NCT03608501	Ixazomib, Thalidomide, Dexamethasone	Non-randomized	Non-US	2019	N	NA
NCT04649060	Melphalan Flufenamide, Dexamethasone, Daratumumab	Randomized	Non-US	2020	N	NA
NCT03196414	CD138/CD19/BCMA CAR-T	Non-randomized	Non-US	2016	N	NA
NCT03277105	Daratumumab	Randomized	Multicenter including US	2017	N	NA
NCT02757326	ABC294640	Non-randomized	US	2016	N	NA
NCT03173430	Blinatumomab	Non-randomized	US	2017	N	NA
NCT03375567	Guided Lesion Biopsies, Standard Lesion Biopsies	Non-randomized	US	2018	N	NA
NCT03768960	Daratumumab	Non-randomized	Non-US	2019	N	NA
NCT02951819	Daratumumab, Cyclophosphamide, Bortezomib, Dexamethasone	Non-randomized	US	2016	N	NA
NCT03070327	EGFRt/BCMA-41Bz CAR-T, Cyclophosphamide, Lenalidomide	Non-randomized	US	2017	N	NA
NCT03475628	Daratumumab	Non-randomized	Non-US	2018	N	NA
NCT03221634	Pembrolizumab, Daratumumab	Non-randomized	Multicenter including US	2017	N	NA
NCT02812706	Isatuximab	Non-randomized	Non-US	2016	N	NA
NCT02516696	Clarithromycin, Lenalidomide, Dexamethasone	Randomized	US	2016	N	NA
NCT02722941	Panobinostat	Non-randomized	US	2016	N	NA
NCT03312530	Cobimetinib, Venetoclax, Atezolizumab	Randomized	Non-US	2017	N	NA

NCT03570983	Melphalan, Carmustine, Etoposide, Cytarabine, Allopurinol	Randomized	US	2018	N	NA
NCT03215030	TAK-573, Dexamethasone	Non-randomized	US	2017	N	NA
NCT02902965	Ibrutinib, Bortezomib, Dexamethasone	Non-randomized	Non-US	2016	N	NA
NCT02906332	Pembrolizumab, Lenalidomide, Dexamethasone	Non-randomized	US	2016	N	NA
NCT03234972	Daratumumab, Bortezomib, Dexamethasone	Randomized	Non-US	2017	N	NA
NCT03068351	RO6870810, Daratumumab	Non-randomized	US	2017	N	NA
NCT04142619	UCARTCS1A	Non-randomized	US	2019	N	NA
NCT03357952	Daratumumab, JNJ-63723283	Randomized	Non-US	2017	N	NA
NCT02661022	SL-401, Pomalidomide, Dexamethasone	Non-randomized	US	2016	N	NA
NCT02726581	Nivolumab, Elotuzumab, Pomalidomide, Dexamethasone	Randomized	Multicenter including US	2016	N	NA
NCT02897830	Ixazomib, Lenalidomide, Dexamethasone	Non-randomized	Non-US	2016	N	NA
NCT02916420	Pomalidomide, Dexamethasone	Non-randomized	Non-US	2016	N	NA
NCT02811978	Bortezomib, Dexamethasone	Randomized	Non-US	2016	N	NA
NCT02843074	Elotuzumab, Lenalidomide, Dexamethasone, Autologous Stem Cell Transplant	Non-randomized	US	2016	N	NA
NCT02977494	Daratumumab, Bortezomib, Dexamethasone	Non-randomized	Non-US	2016	N	NA
NCT02597062	Carfilzomib, Cyclophosphamide, Dexamethasone	Non-randomized	Non-US	2016	N	NA
NCT04398745	Belantamab Mafodotin	Non-randomized	US	2020	N	NA
NCT02410694	Ixazomib, Thalidomide, Dexamethasone	Non-randomized	Non-US	2015	N	NA
NCT02467647	Thalidomide, HLJDT	Randomized	Non-US	2015	N	NA
NCT02852837	Daratumumab	Non-randomized	Non-US	2016	N	NA
NCT03173092	Ixazomib, Lenalidomide, Dexamethasone	Non-randomized	US	2017	N	NA
NCT03710915	HG146	Non-randomized	Non-US	2018	N	NA
NCT03217812	Bortezomib, Melphalan, Prednisone, Daratumumab	Randomized	Non-US	2017	N	NA
NCT02654132	Elotuzumab, Pomalidomide, Dexamethasone	Randomized	Multicenter including US	2016	N	NA
NCT02335983	Carfilzomib, Lenalidomide, Dexamethasone	Non-randomized	US	2015	N	NA
NCT02579863	Pembrolizumab, Lenalidomide, Dexamethasone	Randomized	Multicenter	2015	N	NA

			including US			
NCT02918331	Daratumumab, Lenalidomide, Dexamethasone	Non-randomized	Non-US	2016	N	NA
NCT02412878	Carfilzomib, Dexamethasone	Randomized	Multicenter including US	2015	N	NA
NCT02400242	ACY-241, Pomalidomide, Dexamethasone	Non-randomized	Multicenter including US	2015	N	NA
NCT02461888	Ixazomib, Cyclophosphamide, Dexamethasone	Randomized	Non-US	2015	N	NA
NCT03196947	APO010	Non-randomized	Non-US	2017	N	NA
NCT02332850	Isatuximab, Carfilzomib, Dexamethasone	Randomized	US	2015	N	NA
NCT02197221	Bortezomib, Melphalan	Randomized	Non-US	2015	N	NA
NCT02112045	Granix, Melphalan, Autologous Stem Cell Transplant	Randomized	US	2015	N	NA
NCT02612779	Elotuzumab, Pomalidomide, Dexamethasone, Nivolumab	Randomized	US	2015	N	NA
NCT04434469	RO7297089	Non-randomized	Non-US	2020	N	NA
NCT03361306	Elotuzumab, Carfilzomib, Lenalidomide, Dexamethasone	Non-randomized	US	2017	N	NA
NCT02431208	Atezolizumab,Daratumumab, Lenalidomide, Pomalidomide, Dexamethasone	Non-randomized	US	2015	N	NA
NCT02998047	Lintuzumab-AC225	Non-randomized	US	2016	N	NA
NCT03731832	Pomalidomide, Ixazomib, Dexamethasone, Cyclophosphamide	Non-randomized	Non-US	2018	N	NA
NCT04063189	Clarithromycin, Lenalidomide, Dexamethasone	Non-randomized	Non-US	2017	N	NA
NCT03618602	Bisthianostat	Non-randomized	Non-US	2018	N	NA
NCT02412228	Ixazomib, Cyclophosphamide, Dexamethasone	Non-randomized	US	2015	N	NA
NCT04348006	Bortezomib	Non-randomized	Non-US	2020	N	NA
NCT02211014	Acalabrutinib	Non-randomized	US	2015	N	NA
NCT04505813	NEXI-002 T Cells	Non-randomized	US	2020	N	NA
NCT03448978	Descartes-08	Non-randomized	US	2018	N	NA
NCT03136653	MP0250, Bortezomib, Dexamethasone	Non-randomized	Non-US	2017	N	NA
NCT03697629	Daratumumab	Non-randomized	Non-US	2018	N	NA
NCT03952091	TJ202, Lenalidomide, Dexamethasone	Randomized	Non-US	2019	N	NA
NCT02586038	Ixazomib, Dexamethasone, Cyclophosphamide, Thalidomide	Randomized	Non-US	2015	N	NA

NCT02898064	External Spinal Brace	Randomized	Non-US	2016	N	NA
NCT03589222	Selinexor, Daratumumab, Bortezomib, Dexamethasone	Non-randomized	Non-US	2018	N	NA
NCT03836014	Daratumumab, Lenalidomide, Dexamethasone	Randomized	Non-US	2019	N	NA
NCT03702725	Ibrutinib, Lenalidomide, Dexamethasone	Non-randomized	US	2019	N	NA
NCT03068637	Carevive CPS	Non-randomized	US	2016	N	NA
NCT02447055	Tocilizumab, Melphalan, Fludarabine, Cyclophosphamide, Tacrolimus, Mycophenolate mofetil, Filgrastim, Allogeneic Stem Cell Transplant	Non-randomized	US	2015	N	NA
NCT03934684	Carfilzomib, Dexamethasone, Lenalidomide	Non-randomized	Non-US	2019	N	NA
NCT04635735	Ipilimumab	Non-randomized	US	2020	N	NA
NCT04626752	BCMA CAR-T	Non-randomized	Non-US	2020	N	NA
NCT02336815	Selinexor, Dexamethasone	Non-randomized	Multicenter including US	2015	N	NA
NCT03559764	BCMA CAR-T	Non-randomized	Non-US	2018	N	NA
NCT04613557	CYAD-211, Cyclophosphamide, Fludarabine	Non-randomized	Multicenter including US	2020	N	NA
NCT03742297	Lenalidomide, Carfilzomib, Bortezomib, Daratumumab, Dexamethasone, Prednisone, Melphalan	Randomized	Non-US	2018	N	NA
NCT03674463	LCAR-B4822M-02 CAR-T	Non-randomized	Non-US	2018	N	NA
NCT03187223	Melphalan, Bendamustine	Randomized	Non-US	2017	N	NA
NCT04271644	BCMA CAR-T	Non-randomized	Non-US	2019	N	NA
NCT04272151	BCMA CAR-T	Non-randomized	Non-US	2019	N	NA
NCT03815383	C-CAR088	Non-randomized	Non-US	2019	N	NA
NCT02274519	Tai Chi, Educational Control	Randomized	US	2015	N	NA
NCT03751293	C-CAR088	Non-randomized	Non-US	2019	N	NA
NCT02619812	Colesevelam, Serum-derived bovine immunoglobulin/protein isolate, Double placebo	Randomized	US	2016	N	NA
NCT03713294	Elotuzumab, Pomalidomide, Dexamethasone	Non-randomized	US	2018	N	NA
NCT04601935	LCAR-BCX Cells	Non-randomized	Non-US	2020	N	NA
NCT03716856	BCMA CAR-T	Non-randomized	Non-US	2018	N	NA

NCT04171843	PBCAR269A	Non-randomized	US	2020	N	NA
NCT02286830	Zoledronic Acid	Randomized	Non-US	2015	N	NA
NCT03380039	BCMA CAR-T	Non-randomized	Non-US	2017	N	NA
NCT03318861	KITE-585	Non-randomized	US	2017	N	NA
NCT04662099	CS1/BCMA CAR-T	Non-randomized	Non-US	2020	N	NA
NCT03792763	Denosumab, Placebo	Randomized	Non-US	2019	N	NA
NCT02372240	VLX1570, Dexamethasone	Non-randomized	US	2015	N	NA
NCT03464916	anti-CD38 CAR-T	Non-randomized	US	2018	N	NA
NCT03512353	Carfilzomib, Dexamethasone	Non-randomized	US	2018	N	NA
NCT03029234	Carfilzomib, Dexamethasone	Non-randomized	Non-US	2017	N	NA
NCT04017130	TAK-169	Non-randomized	US	2020	N	NA
NCT04392648	TAK-573, Pomalidomide, Bortezomib, Cyclophosphamide, Dexamethasone	Non-randomized	Multicenter including US	2020	N	NA
NCT03672318	CAR138 T Cells	Non-randomized	US	2019	N	NA
NCT03170882	Ixazomib, Pomalidomide, Dexamethasone	Randomized	Multicenter including US	2017	N	NA
NCT03140943	Carfilzomib, Thalidomide, Dexamethasone	Non-randomized	Non-US	2017	N	NA
NCT03891914	18F-FCH, 18F-FDG	Non-randomized	Non-US	2019	N	NA
NCT04589286	Pack Health's Digital Life Coaching, Electronic Handouts	Randomized	US	2020	N	NA
NCT02831686	Selinexor, Ixazomib, Dexamethasone	Non-randomized	US	2016	N	NA
NCT04405167	Tasquinimod, Ixazomib, Lenalidomide, Dexamethasone	Non-randomized	US	2020	N	NA
NCT02917941	Ixazomib, Lenalidomide, Dexamethasone	Non-randomized	Non-US	2016	N	NA
NCT02992483	MIK665	Non-randomized	Multicenter including US	2017	N	NA
NCT03266692	ACTR087, SEA-BCMA	Non-randomized	US	2018	N	NA
NCT02375555	Bortezomib, Lenalidomide, Elotuzumab, Dexamethasone, Stem Cell Mobilization	Non-randomized	US	2015	N	NA
NCT03242460	Pomalidomide, Cyclophosphamide, Dexamethasone	Non-randomized	Non-US	2015	N	NA
NCT03732703	Abemaciclib, Dexamethasone, Ixazomib, Pomalidomide, Enasidenib, Cobimetinib, Erdafitinib, Venetoclax, Daratumumab	Non-randomized	US	2019	N	NA

NCT03634800	Nivolumab, Radiation Therapy	Non-randomized	US	2018	N	NA
NCT03672253	BCMA CAR-T	Non-randomized	Non-US	2018	N	NA
NCT03450057	Daratumumab	Non-randomized	Non-US	2018	N	NA
NCT03661554	BCMA Nano Antibody CAR-T	Non-randomized	Non-US	2018	N	NA
NCT02579824	DS-3032b	Non-randomized	US	2016	N	NA
NCT03556332	Carfilzomib, Lenalidomide, Dexamethasone, Daratumumab, Autologous Stem Cell Transplant	Non-randomized	US	2018	N	NA
NCT02468687	N-methyl-pyrrolidone	Non-randomized	Non-US	2015	N	NA
NCT03201250	Cabozantinib	Non-randomized	US	2018	N	NA
NCT02426723	CWP232291, Lenalidomide, Dexamethasone	Non-randomized	Multicenter including US	2015	N	NA
NCT02495922	Elotuzumab, Lenalidomide, Bortezomib, Dexamethasone	Randomized	Non-US	2015	N	NA
NCT02952573	JNJ-42756493, Dexamethasone	Non-randomized	Non-US	2017	N	NA
NCT03958656	Anti-SLAMF7 CAR-T	Non-randomized	US	2019	N	NA
NCT02609230	ONC201	Non-randomized	US	2015	N	NA
NCT02401295	ATRA, Celecoxib, Itraconazole	Non-randomized	US	2015	N	NA
NCT03003728	Elotuzumab, Melphalan, Autologous Stem Cell Transplant, Expanded Natural Killer Cells, ALT-803	Non-randomized	US	2019	N	NA
NCT02958969	Apixaban	Non-randomized	US	2018	N	NA
NCT03439280	TAK-079, Pomalidomide, Dexamethasone	Non-randomized	US	2018	N	NA
NCT03602612	Anti-BCMA CAR-T	Non-randomized	US	2018	N	NA
NCT04094961	Ixazomib, Pomalidomide, Dexamethasone	Non-randomized	US	2019	N	NA
NCT03832127	18F-Fludarabine PET	Non-randomized	Non-US	2019	N	NA
NCT03283046	Nivolumab, Lenalidomide, Ipilimumab, Dexamethasone	Non-randomized	US	2017	N	NA
NCT03398200	Hyperbaric Oxygen Therapy	Randomized	US	2018	N	NA
NCT04162353	BCMA-CD19 cCAR T Cells	Non-randomized	Non-US	2019	N	NA
NCT03191981	Cyclophosphamide, Lenalidomide, Pembrolizumab	Non-randomized	Non-US	2017	N	NA
NCT03135925	Supervised Exercise Program	Non-randomized	Non-US	2016	N	NA
NCT02542657	Clarithromycin, Dexamethasone, Ixazomib, Pomalidomide	Non-randomized	US	2015	N	NA

NCT04075721	M3258, Dexamethasone	Non-randomized	Multicenter including US	2019	N	NA
NCT03150316	CKD-581, Lenalidomide, Dexamethasone	Non-randomized	Non-US	2017	N	NA
NCT03051841	CKD-581, Bortezomib, Dexamethasone	Non-randomized	Non-US	2017	N	NA
NCT03436342	68Ga-Pentixafor PET/CT	Non-randomized	Non-US	2019	N	NA
NCT04530812	Kefir	Randomized	US	2020	N	NA
NCT02334865	SVN53-67/M57-KLH Peptide Vaccine, Lenalidomide, Sargramostim, Incomplete Freund's Adjuvant	Non-randomized	US	2016	N	NA
NCT03151811	Melphalan Flufenamide, Pomalidomide, Dexamethasone	Randomized	Multicenter including US	2017	N	NA
NCT02834364	Encorafenib, Binimetinib	Non-randomized	Non-US	2016	N	NA
NCT04100044	Exercise Counseling	Non-randomized	US	2019	N	NA
NCT03793907	FitBit Strength Training	Non-randomized	US	2019	N	NA
NCT04166565	Daratumumab, Bortezomib, Cyclophosphamide, Dexamethasone	Non-randomized	Non-US	2019	N	NA
NCT02978235	TAS4464	Non-randomized	US	2017	N	NA
NCT04000282	SAR442085	Non-randomized	Multicenter including US	2019	N	NA
NCT04407442	Azacitidine, Daratumumab, Dexamethasone	Non-randomized	US	2020	N	NA
NCT03770260	Ixazomib, Pevonedistat	Non-randomized	US	2019	N	NA
NCT02909036	Melphalan, Pegfilgrastim, Autologous Stem Cell Transplant	Non-randomized	US	2016	N	NA
NCT03829020	Bortezomib, Metformin Hydrochloride, Nelfinavir Mesylate	Non-randomized	US	2019	N	NA
NCT03333746	Lenalidomide, Nivolumab	Non-randomized	US	2018	N	NA
NCT03605719	Carfilzomib, Dexamethasone, Nivolumab, Pelareorep	Non-randomized	US	2018	N	NA
NCT03194867	Isatuximab, Cemiplimab	Randomized	Multicenter including US	2018	N	NA
NCT02986451	Clarithromycin, Lenalidomide, Dexamethasone	Non-randomized	Non-US	2016	N	NA
NCT03457142	Abatacept, Dexamethasone, Ixazomib	Non-randomized	US	2018	N	NA
NCT03798678	Carfilzomib, Dexamethasone, Telaglenastat Hydrochloride	Non-randomized	US	2018	N	NA
NCT02971410	Simvastatin	Non-randomized	US	2017	N	NA

NCT03023527	Nivolumab, Pomalidomide, Elotuzumab, Dexamethasone	Non-randomized	Non-US	2017	N	NA
NCT03506360	Ixazomib Citrate, Pembrolizumab, Dexamethasone	Non-randomized	US	2018	N	NA
NCT03733691	Ixazomib, Lenalidomide	Randomized	US	2019	N	NA
NCT03015792	Ibrutinib, Lenalidomide, Dexamethasone	Non-randomized	US	2017	N	NA
NCT03622775	Daratumumab	Non-randomized	US	2019	N	NA
NCT03710421	CS-1 CAR T	Non-randomized	US	2019	N	NA
NCT03338972	FCARH143	Non-randomized	US	2017	N	NA
NCT03502577	BCMA CAR-T, JSMD194	Non-randomized	US	2018	N	NA
NCT02880228	Pembrolizumab, Lenalidomide, Dexamethasone	Non-randomized	US	2016	N	NA
NCT02509052	Leflunomide	Non-randomized	US	2015	N	NA
NCT02514668	Isatuximab	Non-randomized	Multicenter including US	2015	N	NA
NCT02569320	AR-42, Pomalidomide, Dexamethasone	Non-randomized	US	2016	N	NA
NCT03143049	Pomalidomide, Cyclophosphamide, Dexamethasone	Randomized	Non-US	2017	N	NA
NCT02514382	Wild-type Reovirus, Bortezomib, Dexamethasone	Non-randomized	US	2015	N	NA
NCT03256045	Panobinostat, Carfilzomib, Dexamethasone	Non-randomized	US	2018	N	NA
NCT04277845	Lenalidomide, Bortezomib, Dexamethasone	Randomized	Non-US	2020	N	NA
NCT04650724	BCMA CAR-T	Non-randomized	Non-US	2018	N	NA
NCT02697344	R-(-)-Gossypol Acetic Acid, Lenalidomide, Dexamethasone	Non-randomized	US	2017	N	NA
NCT04412889	BCMA/CD19 Dual-target CAR-T	Non-randomized	Non-US	2020	N	NA
NCT04500431	CD269 CAR-T	Non-randomized	Non-US	2018	N	NA
NCT04052880	Daratumumab, Bortezomib, Lenalidomide, Dexamethasone, Ixazomib	Non-randomized	US	2019	N	NA
NCT04217967	Ixazomib, Lenalidomide	Non-randomized	Non-US	2020	N	NA
NCT01415882	Ixazomib, Cyclophosphamide, Dexamethasone	Randomized	US	2016	N	NA
NCT03315026	Siltuximab	Non-randomized	US	2017	N	NA
NCT02082405	Bortezomib, Dexamethasone, Cyclophosphamide	Non-randomized	US	2015	N	NA
NCT03809780	Lenalidomide, Dexamethasone	Non-randomized	Non-US	2019	N	NA
NCT03605056	Chidamide, Lenalidomide, Dexamethasone	Non-randomized	Non-US	2018	N	NA

NCT03752541	BCMA-UCART	Non-randomized	Non-US	2019	N	NA
NCT03481556	Melphalan flufenamide, Dexamethasone, Bortezomib, Daratumumab	Non-randomized	Multicenter including US	2018	N	NA
NCT03428373	ASA, Rivaroxaban	Randomized	Non-US	2019	N	NA
NCT03215524	Daratumumab, Dexamethasone, Cyclophosphamide, Pomalidomide	Randomized	Non-US	2017	N	NA
NCT02751255	Daratumumab, ATRA	Non-randomized	Non-US	2016	N	NA
NCT02578121	Ixazomib, Pomalidomide, Dexamethasone	Non-randomized	US	2015	N	NA
NCT02499081	Ixazomib	Non-randomized	US	2015	N	NA
NCT03246529	BL-8040, G-CSF, Placebo	Randomized	US	2018	N	NA
NCT02577783	Doxorubicin, Bortezomib, Dexamethasone, Hydrochloride Iposome	Randomized	Non-US	2015	N	NA
NCT02140840	Trametinib	Non-randomized	US	2015	N	NA
NCT02272803	Lenalidomide, Dexamethasone, Elotuzumab	Randomized	Non-US	2015	N	NA
NCT03442673	Vinorelbine, Gemcitabine, G-CSF	Randomized	Non-US	2018	N	NA
NCT02192775	MV-NIS	Non-randomized	US	2015	N	NA
NCT03004287	Carfilzomib, Thalidomide, Dexamethasone, Daratumumab, Cisplatin, Adriamycin, Cyclophosphamide, Etoposide, Melphalan, Lenalidomide, Bortezomib, Autologous Stem Cell transplant	Non-randomized	US	2017	N	NA
NCT03000634	Elotuzumab, Bortezomib, Lenalidomide, Dexamethasone	Randomized	US	2017	N	NA
NCT03908138	Lenalidomide, Doxorubicin, Dexamethasone, Bortezomib	Randomized	Non-US	2019	N	NA
NCT03664661	BCMA CAR-T	Non-randomized	Non-US	2018	N	NA
NCT04303091	Physical Activity for Advanced Cancer Treatment	Non-randomized	Non-US	2018	N	NA
NCT04352205	Bortezomib, Daratumumab, Dexamethasone, Lenalidomide, Thalidomide	Non-randomized	US	2020	N	NA
NCT03706547	anti-CD19/BCMA CAR-T	Non-randomized	Non-US	2018	N	NA
NCT03946332	Physical exercises, Optional physical exercises	Randomized	Non-US	2019	N	NA
NCT03574454	Machine Learning in WB-MRI scans	Non-randomized	Non-US	2018	N	NA
NCT03411031	Elotuzumab, Lenalidomide, Dexamethasone	Randomized	US	2018	N	NA
NCT03399539	Dexamethasone, Ixazomib, Venetoclax	Non-randomized	US	2018	N	NA
NCT02619682	Ixazomib, Lenalidomide	Non-randomized	US	2015	N	NA

NCT02492750	Anakinra, Dexamethasone, Lenalidomide, Placebo	Randomized	US	2016	N	NA
NCT03440411	Pomalidomide, Cyclophosphamide, Dexamethasone	Randomized	Non-US	2016	N	NA
NCT02420860	Elotuzumab, Lenalidomide	Non-randomized	US	2015	N	NA
NCT03856112	Dexamethasone, Ixazomib, Venetoclax	Randomized	US	2019	N	NA
NCT02633059	Dexamethasone, Idasanutlin, Ixazomib Citrate	Non-randomized	US	2015	N	NA
NCT03601624	Pomalidomide, Cyclophosphamide, Dexamethasone	Non-randomized	Non-US	2018	N	NA
NCT04178902	ABBV-467	Non-randomized	Multicenter including US	2020	N	NA
NCT03870451	VascuTherm5 Vascular Compression Device	Non-randomized	US	2019	N	NA
NCT04099901	Anakinra, Placebo	Randomized	Non-US	2019	N	NA
NCT02538198	Lenalidomide	Non-randomized	US	2015	N	NA
NCT03233776	Anakinra	Non-randomized	Non-US	2017	N	NA
NCT03641456	Bortezomib, Lenalidomide, Dexamethasone	Non-randomized	Non-US	2020	N	NA
NCT03715478	GSK2857916, Pomalidomide, Dexamethasone	Non-randomized	Non-US	2018	N	NA
NCT02504359	Allogeneic Stem Cell Transplant, Carmustine, Cytarabine, Etoposide, Ixazomib, Melphalan, Methotrexate, Tacrolimus	Non-randomized	US	2015	N	NA
NCT02784483	Atezolizumab	Non-randomized	US	2017	N	NA
NCT03311828	64Cu-DOTA-Daratumumab PET	Non-randomized	US	2018	N	NA
NCT04065308	Dexamethasone, Cyclophosphamide, Etoposide, Cisplatin, Daratumumab	Non-randomized	Non-US	2019	N	NA
NCT02322320	Lenalidomide	Non-randomized	US	2015	N	NA
NCT03506802	18-FHBG, Aldesleukin, CT, Filgrastim, Lenalidomide, Leukapheresis, Melphalan, Plerixafor, PET, NY-ESO-1 CTR PBMCs	Non-randomized	US	2018	N	NA
NCT04275583	TQB3602	Non-randomized	Non-US	2020	N	NA
NCT04028115	Ixazomib	Non-randomized	Non-US	2019	N	NA
NCT03940833	BCMA CAR-NK 92 Cells	Non-randomized	Non-US	2019	N	NA
NCT03741127	P-BCMA-101	Non-randomized	US	2018	N	NA
NCT03267888	Pembrolizumab, Radiation therapy	Non-randomized	US	2018	N	NA
NCT03389347	High Throughput Screening for Drug Sensitivity	Non-randomized	US	2018	N	NA

NCT02719613	Elotuzumab, Dexamethasone, Lenalidomide, Bortezomib, Pomalidomide, Nivolumab	Non-randomized	Multicenter including US	2016	N	NA
NCT02716805	Tremelimumab, Durvalumab, Prevnar-13, Melphalan	Non-randomized	US	2016	N	NA
NCT03858205	Low-Dose Radiotherapy	Non-randomized	US	2019	N	NA
NCT02420223	Propanolol	Randomized	US	2015	N	NA
NCT03184194	Nivolumab, Daratumumab, Cyclophosphamide	Randomized	Non-US	2018	N	NA
NCT02944565	Daratumumab	Non-randomized	US	2017	N	NA
NCT03683277	Ixazomib, Pomalidomide, Dexamethasone	Non-randomized	Non-US	2018	N	NA
NCT03031730	Carfilzomib, Dexamethasone, Dexamethasone Sodium Phosphate, Lenalidomide, KRT-232	Non-randomized	US	2017	N	NA
NCT03143036	Daratumumab, Thalidomide, Dexamethasone	Non-randomized	Non-US	2018	N	NA
NCT04150965	Elotuzumab, Pomalidomide, Dexamethasone, Anti-LAG-3, Anti-TIGIT	Randomized	US	2020	N	NA
NCT02283775	Isatuximab, Pomalidomide, Dexamethasone	Non-randomized	US	2015	N	NA
NCT03619252	PCV13, Standard Antibacterial Prophylaxis	Randomized	Non-US	2018	N	NA
NCT02749617	Apixaban, Dexamethasone	Non-randomized	Non-US	2016	N	NA
NCT04184050	HPN217	Non-randomized	Multicenter including US	2020	N	NA
NCT04065789	Carfilzomib, Daratumumab, Lenalidomide, Dexamethasone	Non-randomized	Non-US	2018	N	NA
NCT02158052	Tacrolimus, Equine ATG, Kidney Transplant, Bone Marrow Transplant, TBI	Non-randomized	US	2015	N	NA
NCT03385096	Busulfan, Cyclophosphamide, Etoposide, Melphalan	Randomized	Non-US	2018	N	NA
NCT04106700	Apixaban	Non-randomized	Non-US	2019	N	NA
NCT04080531	Pneumococcal 13-valent Conjugate Vaccine, Trivalent Influenza Vaccine	Randomized	US	2019	N	NA
NCT03168438	Letetresgene autoleucel, Pembrolizumab, Fludarabine, Cyclophosphamide	Non-randomized	US	2017	N	NA
NCT03399799	Talquetamab	Non-randomized	Multicenter including US	2017	N	NA
NCT04008888	PI, IMids, Dexamethasone, Fludarabine, Melphalan, Autologous Stem Cell Transplant, Allogeneic Stem Cell Transplant	Non-randomized	Non-US	2018	N	NA
NCT03145181	Teclistamab	Non-randomized	Multicenter including US	2017	N	NA

NCT03959358	Lenalidomide, Pomalidomide	Non-randomized	Non-US	2020	N	NA
NCT03767257	Colesevelam Pill	Non-randomized	US	2018	N	NA
NCT03225417	Ixazomib, Tacrolimus, Sirolimus	Randomized	Non-US	2017	N	NA
NCT04268199	Bortezomib	Non-randomized	Non-US	2020	N	NA
NCT03078452	Power drill, Jamshidi needle	Randomized	US	2017	N	NA
NCT02700841	Tetanus Toxoid Vaccine, T-Cell Depleted Hematopoietic Stem Cell Transplant, Melphalan	Randomized	US	2016	N	NA
NCT02765854	Ixazomib, Lenalidomide, Dexamethasone	Randomized	US	2016	N	NA
NCT02566265	Fluzone High Dose Vaccine, Placebo	Randomized	US	2015	N	NA
NCT03198754	PEI Experimental Light, Comparison Light	Randomized	US	2016	N	NA
NCT04677452	JWCAR129	Non-randomized	Non-US	2020	N	NA
NCT03639610	Melphalan flufenamide, Dexamethasone	Non-randomized	Non-US	2018	N	NA
